# Supplementary material for: Combining Human Genetics of Multiple Sclerosis with Oxidative Stress Phenotype for Drug Repositioning
Source: Pharmaceutics. 2021 Dec 2;13(12):2064. doi: 10.3390/pharmaceutics13122064 (PMC8705550; doi:10.3390/pharmaceutics13122064)
Supplement: Supplementary file 1 [file pharmaceutics-13-02064-s001.zip › pharmaceutics-1442358- Supplementary Materials Proofreading.pdf]

pharmaceutics

Supplementary Materials: Combining Human Genetics of Multiple Sclerosis with Oxidative Stress Phenotype for Drug Repositioning

Stefania Olla, Maristella Steri, Alessia Formato Michael B. Whalen, Silvia Corbisiero and Cristina Agres

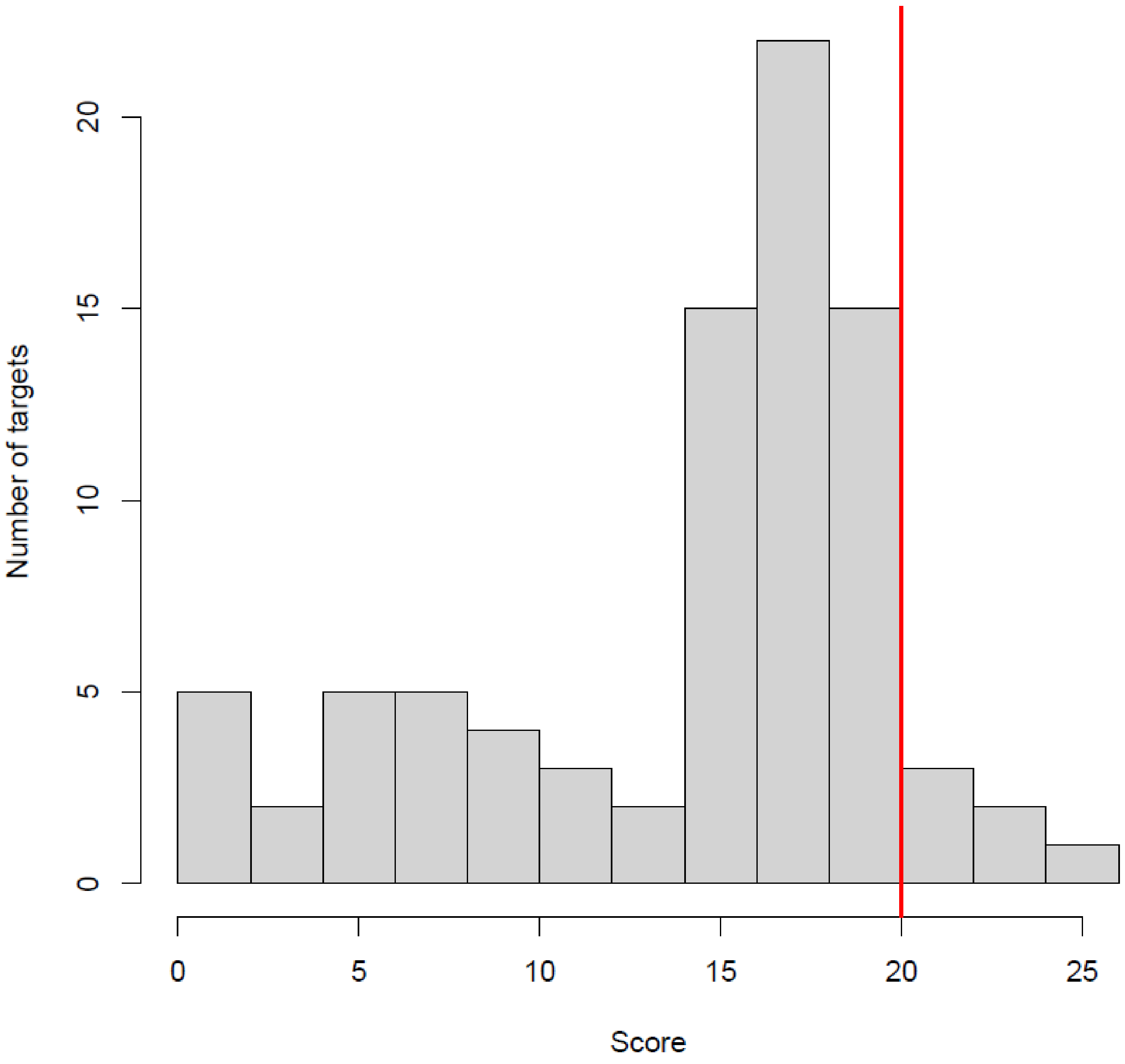

Figure S1. Prioritization score distribution
